# A phase Ib/II study of modakafusp alfa alone and in combination with pembrolizumab in patients with advanced or metastatic solid tumors

**DOI:** 10.3389/fonc.2025.1620987

**Published:** 2025-12-08

**Authors:** David Gill, Charles L. Cowey, Gregory A. Daniels, David Sommerhalder, Raghad Abdul-Karim, John M. Kirkwood, Joanna Kolodney, Inderjit Mehmi, Rachel Roberts-Thomson, James Strauss, Sajeve Thomas, Eric Whitman, Yan Xing, Meredith McKean, Sabrina Collins, Cheryl Li, Gurpanna Saggu, Tian Chen, Shining Wang, Marina Lewis, Xavier Parot, Melissa Johnson

**Affiliations:** 1 Intermountain Health, Salt Lake City, UT, United States; 2 Baylor Charles A. Sammons Cancer Center, Texas Oncology, Dallas, TX, United States; 3 Department of Medicine, Division of Hematology Oncology, University of California San Diego, La Jolla, CA, United States; 4 NEXT Oncology, San Antonio, TX, United States; 5 Nova Oncology, McAllen, TX, United States; 6 Medicine, UPMC Hillman Cancer Center and the University of Pittsburgh, Pittsburgh, PA, United States; 7 Medical Oncology, West Virginia University, Morgantown, WV, United States; 8 The Angeles Clinic and Research Institute, CS Cancer, Los Angeles, CA, United States; 9 Medical Oncology, The Queen Elizabeth Hospital, Adelaide, SA, Australia; 10 Mary Crowley Cancer Research, Dallas, TX, United States; 11 Medical Oncology, Advent Health, Orlando, FL, United States; 12 Oncology, Atlantic Health System, Morristown, NJ, United States; 13 Medical Oncology, City of Hope Comprehensive Cancer Center, Duarte, CA, United States; 14 Sarah Cannon Research Institute, Nashville, TN, United States; 15 Oncology Precision and Translational Medicine, Takeda Development Center Americas, Inc. (TDCA), Cambridge, MA, United States; 16 Quantitative Clinical Pharmacology, Takeda Development Center Americas, Inc. (TDCA), Cambridge, MA, United States; 17 Precision and Translation Medicine, Oncology Therapeutic Area Unit, Takeda Development Center Americas, Inc. (TDCA), Cambridge, MA, United States; 18 Oncology, Takeda Development Center Americas, Inc. (TDCA), Cambridge, MA, United States; 19 Oncology Clinical Science, Takeda Development Center Americas, Inc. (TDCA), Cambridge, MA, United States

**Keywords:** modakafusp alfa, pembrolizumab, solid tumors, immunocytokine, innate immunity activation

## Abstract

**Background:**

Modakafusp alfa is a novel immunocytokine comprising two attenuated interferon-α2b molecules fused to an anti-CD38 IgG4 monoclonal antibody. Modakafusp alfa has shown immune cell activation and antitumor activity in preclinical mouse models, including in combination with an anti-programmed cell death (PD-1) receptor in tumors that do not express CD38, and demonstrated clinical responses and immune activation in patients with relapsed/refractory multiple myeloma.

**Methods:**

In phase Ib, adult patients with advanced/metastatic solid tumors received escalating doses of modakafusp alfa 0.1–1.5 mg/kg intravenously every 3 weeks (Q3W) across six dosing cohorts. In phase II, patients with unresectable/metastatic cutaneous melanoma and resistance to ≤2 anti-PD-1 therapies in the metastatic setting received modakafusp alfa 1 mg/kg Q3W in combination with pembrolizumab Q6W. Primary objectives were to determine the safety/tolerability as a single agent in phase I, and efficacy in combination with pembrolizumab in phase II.

**Results:**

A total of 21 and 24 patients were enrolled across phases Ib and II, respectively. The recommended phase II dose of modakafusp alfa was 1 mg/kg. The most common drug-related adverse events were infusion-related reactions (IRRs; 52.4%) and thrombocytopenia (28.6%) in phase Ib; and headache (58.3%), fatigue (54.2%), IRRs (41.7%), neutropenia (37.5%), and nausea (33.3%) in phase II. In phase Ib, seven patients had a best response of stable disease (SD); in phase II, one patient had a confirmed complete response, one had a confirmed partial response, and seven had SD. All immunogenicity-evaluable patients were anti-drug antibodies (ADAs) positive following treatment with modakafusp alfa; neutralizing ADAs were reported in 82.4% and 90.9% of patients in phases Ib and II, respectively, which was associated with drug exposure reduction. Pharmacodynamic analyses demonstrated innate and adaptive immune activation in peripheral blood and within tumors. Paired biopsy analysis revealed two subgroups of patients defined by differences in CD38 upregulation, accompanied by differential intratumoral pharmacodynamic changes. Correlative analysis was inconclusive.

**Conclusions:**

Modakafusp alfa induces innate and adaptive immune responses, supporting its hypothesized mechanism of action (MoA) in patients with advanced solid tumors. High immunogenicity and the potentially limited treatment effect of the interferon MoA may have contributed to limited efficacy in these patients.

**Clinical trial registration:**

## Introduction

There have been significant advances in the treatment of advanced melanoma over the last decade following introduction of immunotherapies and targeted therapies (1–3). Indeed, these treatments now represent the standard of care for advanced melanoma, with management guidelines recommending a combination of immune checkpoint inhibitors (CPIs), and molecularly targeted treatments with *BRAF/MEK* inhibitors (2–4). Pembrolizumab, one such CPI recommended by guidelines (2, 4) is a programmed cell death (PD-1) inhibitor approved in the United States and Europe for the treatment of patients with unresectable or metastatic melanoma (5, 6). However, not all patients respond to CPI therapy and some patients may develop acquired resistance following an initial response (7, 8).

Historically, interferon-alpha (IFNα) has been an effective adjuvant therapy for melanoma and has improved survival in patients with high-risk disease (9, 10). However, IFNα therapy is associated with toxicities involving constitutional, neurologic, cutaneous, gastrointestinal, and hematologic systems (9).

Modakafusp alfa is a novel immunocytokine composed of two attenuated IFN-α2b molecules fused to an anti-CD38 IgG4 monoclonal antibody (11). Modakafusp alfa binds with high affinity to a unique epitope of CD38 whereas the attenuation of the IFN molecules results in reduced IFNα receptor binding affinity (11). CD38 is a transmembrane glycoprotein with ectoenzymatic activity that is highly and uniformly expressed on multiple myeloma cells and expressed at relatively low levels on normal lymphoid and myeloid cells (12). The binding of modakafusp alfa via CD38 targeting moieties drives targeted IFN type I signaling to innate and adaptive immune cells and elicits direct anti-proliferative/apoptotic signals to tumor cells expressing CD38 (13, 14). Modakafusp alfa was investigated for the treatment of multiple myeloma as a single agent and in combination with the standard of care and showed clinical responses and immune activation in patients with relapsed/refractory multiple myeloma (11, 13).

There is evidence of CD38 expression within the tumor microenvironment of solid tumors, including melanoma (15). CD38 has emerged as a promising therapeutic target in solid tumors due to its role in modulating the tumor microenvironment, which enables dual mechanisms of action involving direct tumor targeting and immune modulation (8, 16). Non-clinical studies evaluating the activity of a murine cross-reactive surrogate of modakafusp alfa (anti-murine CD38 Attenukine™; mCD38-mAtt) in a non-CD38-expressing, mildly IFNα-sensitive CT26 tumor model in immunocompetent mice showed significant antitumor activity posttreatment with mCD38-mAtt versus a non-targeted control, as well as increased CD8 T-cell proliferation (17).

The reduced IFN receptor binding affinity of the attenuated IFN-α2b molecules of modakafusp alfa reduces the potential for off-target binding and toxicity (18), which is a limitation of IFNα treatments historically used in melanoma (9, 19). Given the propensity for tumors to develop resistance to CPIs through mechanisms potentially associated with upregulation of CD38 in tumor cells, which inhibit CD8-positive T-cell function via adenosine receptor signaling (8), and that modakafusp alfa binds with high specificity to a unique epitope of CD38 (13, 14, 20), there is a strong rationale to evaluate modakafusp alfa in combination with pembrolizumab. The antitumor responses in CD38 tumor cells were attributed to both the direct antiproliferative effects of IFNα and the indirect activation of innate immune cells, including M1 macrophages and natural killer (NK) cells (20). Furthermore, prior preclinical research demonstrated antitumor activity with mCD38-mATT combined with an anti-PD-1 in murine tumor models (unpublished data). Based on this rationale, we investigated the safety/tolerability and antitumor activity of modakafusp alfa as a single agent and in combination with pembrolizumab in adults with advanced or metastatic solid tumors.

## Methods

### Trial design and patients

This open-label, phase Ib/II trial consisted of a phase Ib dose escalation of modakafusp alfa as a single agent and a phase II safety lead-in followed by dose expansion in combination with pembrolizumab. Eligible patients were aged ≥18 years, with histologically confirmed locally advanced or metastatic solid tumors not amenable to curative therapy (phase Ib) and unresectable/metastatic cutaneous melanoma (phase II) with either of the following: primary resistance to ≤2 prior lines of anti-PD-1-containing treatments (cohort 1), acquired resistance to ≤2 prior lines of anti-PD-1-containing treatments (cohort 2), or naïve to prior line(s) of anti-PD-1-containing treatment (cohort 3). Patients were required to have an Eastern Cooperative Oncology Group performance status of 0–1 and measurable disease per Response Evaluation Criteria in Solid Tumors (RECIST) version 1.1. A full listing of inclusion and exclusion criteria can be found in the Supplement. The data cutoff date was February 14, 2024.

### Objectives

The primary objectives of the study were to determine the safety/tolerability of modakafusp alfa as a single agent in patients with locally advanced/metastatic solid tumors in phase Ib, and in combination with pembrolizumab in unresectable/metastatic cutaneous melanoma (phase II safety lead-in), and determine the efficacy in combination with pembrolizumab in patients with unresectable/metastatic cutaneous melanoma (phase II expansion). The secondary objectives were to define the maximum tolerated dose (MTD) and/or pharmacologically active dose (PAD) of modakafusp alfa (phase Ib); select the recommended phase II dose (RP2D) alone (phase Ib) and in combination with pembrolizumab (phase II safety lead-in); determine the pharmacokinetics (PK) of modakafusp alfa alone (phase Ib) or in combination with pembrolizumab (phase II safety lead-in); assess preliminary antitumor activity as a single agent and in combination with pembrolizumab per immune-related RECIST criteria (phase Ib and II); and characterize immunogenicity in solid tumors (phase Ib/phase II). Pharmacodynamics were evaluated as an exploratory endpoint.

### Assessments

#### Phase Ib dose escalation

Dose escalation followed a 3 + 3 design to determine either an MTD or PAD (defined by a PK/pharmacodynamic model or exposure–response analysis). In phase Ib, a minimum of three patients were enrolled at a starting dose of modakafusp alfa 0.1 mg/kg administered, as a single agent, every 3 weeks (Q3W). Doses were escalated to 0.2, 0.4, 0.75, 1.0, and 1.5 mg/kg. An intermittent dose level of 1 mg/kg was also evaluated. Patients were assessed for dose-limiting toxicities (DLTs) until cycle 2, day 1. Toxicity was evaluated according to the National Cancer Institute Common Terminology Criteria for Adverse Events version 5 (NCI CTCAE v.5). A DLT was defined as any of the following adverse events (AEs) that occurred in the escalation phase or in the combination safety lead-in phase during cycle 1 unless they were considered by the investigator to be clearly unrelated to therapy with modakafusp alfa:

- Any grade 5 treatment-emergent AE (TEAE).
- Febrile neutropenia: grade ≥3 neutropenia (ANC <1.0 × 10^9^/L) with fever and/or infection, where fever was defined as a single temperature of >38.3°C or sustained temperature of ≥38°C for more than 1 h.
- Grade 4 neutropenia lasting >7 days.
- Grade 4 thrombocytopenia lasting >7 consecutive days, or if platelet transfusion was required, or if grade ≥2 bleeding happened at any moment. A platelet count of <10 × 10^9^/L at any time was a DLT.
- Grade ≥3 thrombocytopenia lasting >14 days or accompanied by grade ≥2 bleeding.
- Any grade 3 immune-related AEs such as pericarditis, pneumonitis, cardiotoxicity, hepatitis, or neurotoxicity.
- Delay in the initiation of cycle 2 by >14 days from the calculated start date due to a lack of adequate recovery of treatment-related hematological or non-hematologic toxicities.
- Any grade ≥3 non-hematologic toxicity with the following exceptions:
  ◼ Grade 3 arthralgia/myalgia that responded to non-steroidal anti-inflammatory drugs within 1 week.
  ◼ Grade 3 fatigue lasting <3 days.
  ◼ Grade 3 endocrine disorder that was managed with or without therapy and the patient was asymptomatic.
  ◼ Grade 3 or 4 inflammatory reaction attributed to a local antitumor response.
  ◼ Grade 3 or 4 asymptomatic laboratory changes (other than renal and hepatic laboratory values) that could be successfully corrected (reversion of grade 4 events to grade ≤2, reversion of grade 3 events to grade ≤1 or baseline) within 72 h.
  ◼ Isolated grade 3 elevation of alanine aminotransferase and/or aspartate aminotransferase that resolved to grade ≤1 or baseline within 7 days.
  ◼ Grade 3 nausea and/or emesis that could be controlled to grade <3 in ≤3 days with the use of optimal antiemetics (defined as an antiemetic regimen that employs both a 5-hydroxytryptamine 3 serotonin receptor antagonist and a corticosteroid given in standard doses and according to standard schedules).
  ◼ Grade 3 rash and pruritus that responded to a standard treatment and resolved or improved to grade <3 within 7 days.
  ◼ Grade 3 diarrhea that could be controlled to grade <3 in ≤3 days with appropriate treatment.
  ◼ Any other grade 3 TEAE for which NCI CTCAE v.5 grade 4 or 5 does not exist or the NCI CTCAE does not consider the grade 4 AE to be life-threatening.

- Any grade 2 non-hematologic toxicity that was considered by the investigator to be related to the study drug and dose-limiting.

In the dose escalation phase, DLTs were defined as events meeting the criteria above that occurred before cycle 2, day 1 administration. TEAEs meeting DLT definitions occurring in later cycles or during expansion determined the suitability of the selected dose as the RP2D.

#### Phase II dose expansion

The phase II dose expansion, in combination with pembrolizumab, was initiated once the RP2D of modakafusp alfa was determined in phase Ib. The combination treatment cohorts started with a single safety lead-in period to evaluate the safety and tolerability of the RP2D for modakafusp alfa in combination with pembrolizumab 400 mg Q6W during the cycle 1 DLT evaluation period. If there were no DLTs reported in the first three patients during this period, the combination dose and regimen could be selected for three disease cohorts: unresectable/metastatic cutaneous melanoma with primary resistance to ≤2 prior lines of anti-PD-1; unresectable/metastatic cutaneous melanoma with acquired resistance to ≤2 prior lines of anti-PD-1; or unresectable/metastatic cutaneous melanoma naïve to prior anti-PD-1-containing treatments in the metastatic setting. A maximum of 25 patients were planned to be enrolled in each of the three cohorts.

### PK assessments

Serum samples for PK measurements were collected at the following timepoints during phase Ib: cycle 1, day 1 (pre-dose, end of infusion [EOI], and at 1, 2, and 6 h post-dose); cycle 1, days 2–4 (at 24, 48, and 72 h post-dose, respectively); cycle 2, day 1 (pre-dose, EOI, and at 1, 2, and 6 h post-dose), cycle 2, days 2–4 (at 24, 48, and 72 h post-dose, respectively); and cycles 3–6, day 1 (pre-dose, EOI, and at 2 h post-dose).

Free concentrations of modakafusp alfa (i.e., concentration of modakafusp alfa with both CD38 and IFN-α-binding site intact) in the serum were determined using a validated enzyme-linked immunosorbent assay. PK data were estimated using non-compartmental methods with Phoenix WinNonlin™. Parameters were estimated from the concentration–time profiles for the PK analysis population. Summary statistics were calculated for the following parameters: maximum serum concentration (C_max_), area under the serum concentration–time curve from time 0 to infinity, area under the serum concentration–time curve from time 0 to time of the last quantifiable concentration (AUC_last_), terminal disposition rate constant, time to C_max_ (t_max_), clearance, apparent volume of distribution at steady state, and apparent terminal elimination phase half-life (t_1/2z_).

### Immunogenicity assessments

Serum samples for immunogenicity testing were collected before dosing on day 1 of each cycle whereas the patient remained on treatment and, if possible, at the end of the treatment follow-up visit. The presence of anti-drug antibodies (ADAs) and ADA titer values were detected using a validated bridging electrochemiluminescence assay. Specifically, samples and controls were acid-treated, neutralized, and incubated with biotin- and ruthenium-labeled modakafusp alfa. ADA bound both labels, and complexes were captured on streptavidin-coated Standard Meso Scale Discovery (MSD) plates (MSD, Rockville, Maryland). Voltage-triggered chemiluminescence from ruthenium in a read buffer, measured as relative light unit, reflected the ADA levels. Samples that screened positive were retested to confirm positivity. Confirmed ADA-positive samples were then titrated utilizing the same assay at various dilutions of serum to determine ADA titer. Additionally, positive ADA samples were further characterized for their binding domain specificity (IgG4IFN or anti-CD38), as well as for their neutralizing capacity. The domain specificity was assessed utilizing labeled domain drugs following the same methodology as the total ADA assay. The neutralizing anti-drug antibodies (NAbs) assay detected modakafusp alfa NAbs in human serum using a cell-based luciferase expression system. The assay uses THP-1 Dual™ cells (InvivoGen, San Diego, CA), which emit luminescence when the interferon regulatory factor (IRF) pathway is activated by modakafusp alfa. NAbs interfere with the ability of modakafusp alfa to signal via the IRF pathway, resulting in reduced signal. The procedure spans 3 days, involving magnetic bead preparation, serum pretreatment, incubation with cells, and luminescence measurement. Samples were considered NAb-positive if the luminescence signal fell below the plate cut point (signal-to-drug concentration ratio [S/DC] ≤0.83). All immunogenicity assays were validated for their intended use.

The proportions of patients with detectable ADAs (transient and persistent, titer and specificity) and NAbs during the study were summarized. The apparent effect of immunogenicity on modakafusp alfa PK was evaluated. Analysis was based on available data from patients with a baseline assessment and/or post-baseline immunogenicity assessment in the safety analysis set. Summaries were provided separately for each study phase and by dose, as applicable. These analyses were exploratory, and all results were descriptive in nature.

### Pharmacodynamic assessments

Pharmacodynamic evaluations, which were all exploratory in nature, were conducted on whole blood, serum, and tumor biopsy samples at a central laboratory.

The following assessments were conducted:

- Fresh whole blood samples were analyzed for CD38 receptor occupancy and density using a two-tube nine-color flow cytometry assay to establish target engagement. Upon analysis, samples were red blood cells lysed with Pharm Lyse buffer, washed in phosphate-buffered saline (PBS) with 0.5% bovine serum albumin (BSA), blocked with Fc Block containing a viability dye, and stained under two conditions. Saturating levels of either isotype control (human IgG4) or modakafusp alfa were added in addition to surface marker antibodies (**Supplementary Table S1**) to enable detection of CD38 receptor occupancy and receptor density on T cells, B cells, monocytes, and NK cells. Samples were then washed with PBS/BSA, stained with anti-IgG4 phycoerythrin (PE), washed, fixed with a 1.6% paraformaldehyde solution in PBS/BSA, and washed into PBS/BSA for analysis using an LSR II flow cytometer (BD Biosciences, San Jose, CA). Analysis of the samples followed the gating strategy in **Supplementary Figure S1**.
- RNA was extracted from whole blood, collected into PAXgene RNA tubes (BD Biosciences, San Jose, CA) and analyzed by whole transcriptome RNA sequencing to generate gene expression data. These data were then analyzed to determine type I IFN pathway activation (type I IFN gene signature score, expressed as average fragments per kilobase per million for the 25-gene IFN-stimulated gene score) (21).
- Serum samples were used for evaluation of systemic cytokine and chemokine concentrations, using Olink Proteomics’ proprietary proximity extension assay technology and their Target 48 panel, following all Olink Proteomic SOPs. The concentrations of the cytokines and chemokines, including monocyte chemoattractant protein (MCP)-2, IFN gamma-induced protein-10, interleukin (IL)-27, IFNγ, IL-15, IL-6, and tumor necrosis factor α were quantified in pg/mL.
- Peripheral blood mononuclear cells were cryopreserved from Pharm Lysed whole blood samples. Upon analysis, samples were thawed, washed, and stained first with the viability dye followed by the surface staining antibodies (**Supplementary Table S2**), with a wash step in between. Samples were then fixed, permeabilized, and stained with the intracellular marker antibodies (**Supplementary Table S2**. Samples were stored in DNA intercalator plus fixative at 4°C. After storage, samples were washed into water for acquisition on the Fluidigm Helios mass cytometer. Analysis of the samples followed the gating strategy in **Supplementary Figure S2**. The assay was developed to characterize changes in immune cell prevalence and activation in whole blood.
- Tumor biopsy samples were stored as formalin-fixed paraffin-embedded (FFPE) blocks; freshly sectioned slides from FFPE blocks were stained using the MultiOmyx Hyperplexed immunofluorescence assay with a 20-marker panel (**Supplementary Table S3**). Multiplex staining was performed in 11 rounds of imaging following a predetermined marker sequence (**Supplementary Table S4**) following MultiOmyx guidelines and SOPs. For each round, stained imaging was acquired for the two antibodies listed for that round, after which bleaching was performed to remove signal prior to staining the next round. Staining specificity and subcellular localization were evaluated for each biomarker used in the panel. Tissue retention was evaluated qualitatively for all samples. Additionally, evaluation was performed to assess if there were any tissue specific artifacts or autofluorescence that might impact signal to noise of each stained biomarker.

### Statistical analysis

The safety analysis set included all enrolled patients who received ≥1 dose of the study drug, including incomplete doses. The response-evaluable analysis set was a subset of the safety analysis set and included patients with measurable disease at baseline and ≥1 posttreatment response assessment. The PK analysis set included patients from the safety analysis set who had sufficient data to calculate the ≥1 PK parameter for the study drug. The immunogenicity-evaluable population included patients with ADAs evaluated at baseline and ≥1 posttreatment visit. The overall response rate (ORR) and disease control rate were summarized using descriptive statistics with 95% confidence intervals.

### Criteria for early termination

A Bayesian predictive probability design was used to allow two futility analyses to stop early for futility for the combination therapy cohorts in phase II, with the investigator-assessed ORR as the endpoint. The first and second futility analyses were planned for when 10 and 15 response-evaluable patients, respectively, had completed ≥1 posttreatment response assessment or had discontinued treatment with modakafusp alfa before a posttreatment response assessment. A cohort with the posterior probability of success <10% would stop enrollment for futility. The second futility analysis could be skipped if all or almost all patients had already been enrolled at the time of the analysis.

### Data availability

The datasets, including the redacted study protocols, redacted statistical analysis plans, and individual participant data supporting the results of the completed study will be made available after the publication of the final study results within 3 months from initial request to researchers who provide a methodologically sound proposal. The data will be provided after its de-identification, in compliance with applicable privacy laws, data protection, and requirements for consent and anonymization.

## Results

### Patient characteristics

Between December 12, 2019, and May 23, 2023, 21 patients had been enrolled in phase Ib, and 24 patients in phase II; all patients were included in the safety analysis set. The phase II expansion study included three patients from the safety lead-in. Of the remaining 21 patients, 9 patients were in cohort 1 (primary resistance) and 12 patients were in cohort 2 (acquired resistance). No patients were enrolled into cohort 3 (naïve).

As of February 14, 2024, all 21 patients in phase Ib had discontinued treatment: 13 due to progressive disease, three due to AEs, three due to patient withdrawal, and two for other reasons (**Supplementary Table S5**). All of the 24 patients enrolled in phase II also discontinued treatment: 17 due to disease progression, 2 each due to patient withdrawal or other reasons, and 1 each due to AEs, death, or symptomatic deterioration. The median (range) follow-ups for phase Ib and phase II were 3.8 (0.8–36.3) and 7.2 (1.6–22.4) months, respectively.

Patient baseline characteristics and demographics for all patients are summarized in **Table 1**. The median ages were 63.0 and 65.0 years in phase Ib and phase II, respectively. At initial diagnosis, 9 of 21 patients in the phase Ib arm had colorectal cancer, whereas in phase II, all 24 patients had melanoma. The median (range) time since initial diagnosis was 3.0 (0.9–13.7) years for phase Ib and 2.7 (1.2–29.3) years for phase II. Patients in phase Ib and phase II had received a median of 3.5 and 2.0 prior lines of therapy, respectively. Among the 24 patients in phase II, 5 (20.8%) had received prior BRAF inhibitors and all had received a prior CPI, per eligibility criteria.

**Table 1:** Baseline demographics and disease characteristics (safety analysis set).

Characteristic	Phase Ib (dose escalation) (n=21)	Phase II (dose expansion) (n=24)
Median age, years (range)	63.0 (42−80)	65.0 (23−78)
Male	12 (57.1)	17 (70.8)
Race, n (%)		
White	18 (85.7)	20 (83.3)
Black or African American	0	1 (4.2)
Asian	0	1 (4.2)
Not reported	3 (14.3)	2 (8.3)
ECOG performance score, n (%)		
0	4 (19.0)	13 (54.2)
1	17 (81.0)	11 (45.8)
Type of cancer at initial diagnosis		
Anal	2 (9.5)	0
Bile duct	1 (4.8)	0
Bone	1 (4.8)	0
Colorectal*	9 (42.9)	0
Cutaneous melanoma	0	3 (12.5)
Gastroesophageal	1 (4.8)	0
Melanoma	2 (9.5)	21 (87.5)
Pancreatic	2 (9.5)	0
Renal	2 (9.5)	0
Uterine	1 (4.8)	0
Median time from initial diagnosis, years (range)	3.0 (0.9−13.7)	2.4 (0.3–29.3)
Disease stage at study entry, n (%)		
III	0	6 (25.0)
IV	21 (100)	17 (70.8)
Other	0	1 (4.2)
Median lines of prior therapy, n (range)	3 (2–7)	2 (1–6)
Prior systemic anticancer therapy, n (%)	20 (95.2)	24 (100)
BRAF inhibitors	4 (19.0)	5 (20.8)
Checkpoint inhibitors	6 (28.6)	24 (100)
Tyrosine kinase inhibitors	4 (19.0)	1 (4.2)

*Includes colon (n=1), colorectal (n=1), and rectal (n=2) cancers.

ECOG, Eastern Cooperative Oncology Group.

### Safety

In phase Ib, DLTs were observed in two patients treated at 1.5 mg/kg (grade 4 thrombocytopenia; grade 3 confusional state) and the RP2D of modakafusp alfa was determined to be 1 mg/kg Q3W (18).

In phase Ib, TEAEs were reported in all patients, including infusion-related reactions (IRRs; 52.4%; all grade 1–2), thrombocytopenia (33.3%; grade ≥3, 9.5%), and neutropenia (28.6%; grade ≥3, 23.8%) (**Table 2**). Drug-related TEAEs were reported in 81.0% of patients; the most common were IRRs (52.4%; all grade 1–2), thrombocytopenia (28.6%; grade ≥3, 4.8%), and neutropenia (14.3%; grade ≥3, 14.3%). Clinical symptoms of IRRs included chills (47.6%), nausea (23.8%), dyspnea, hypoxia, and vomiting (14.3% each); grade 2 cytokine release syndrome was reported in one patient in the 1.5 mg/kg cohort. Grade ≥3 TEAEs were reported in 66.7% of patients; the most common were neutropenia in 23.8% and lymphocyte count decrease in 14.3%. Serious AEs (SAEs) occurred in 61.9% of patients, most of which were IRRs (33.3%). One patient discontinued modakafusp alfa due to confusional state.

**Table 2:** Safety summary and common TEAEs (≥20% in phase Ib or phase II) (safety analysis set).

Category, n (%)	Monotherapy in phase Ib (n=21)	Combination with pembrolizumab in phase II (n=24)
Any TEAE	21 (100)	23 (95.8)
Grade ≥3	14 (66.7)	16 (66.7)
Treatment-related*	17 (81.0)	23 (95.8)
Serious TEAE	13 (61.9)	8 (33.3)
Leading to study drug discontinuation	3 (14.3)	1 (4.2)
On-study deaths^†^	3 (14.3)	2 (8.3)
Most common TEAEs (≥20% in phase Ib or II)		
Fatigue	4 (19.0)	13 (54.2)
Headache	3 (14.3)	14 (58.3)
Infusion-related reactions	11 (52.4)	10 (41.7)
Neutropenia	6 (28.6)	9 (37.5)
Nausea	5 (23.8)	8 (33.3)
Thrombocytopenia	7 (33.3)	7 (29.2)
Leukopenia	2 (9.5)	6 (25.0)
Vomiting	1 (4.8)	6 (25.0)

*Related to modakafusp alfa or pembrolizumab.

^†^Defined as deaths that occurred between the first dose of study drug and up to 30 days after the last dose of study drug and deaths related to study drug that occurred 30 days after the last dose of study drug.

TEAE, treatment-emergent adverse event.

In phase II, no DLTs were reported among the three patients in the safety lead-in cohort, and so patients were enrolled into cohorts 1 and 2 to receive modakafusp alfa 1 mg/kg Q3W in combination with pembrolizumab 400 mg Q6W. Among all 24 patients in phase II, TEAEs occurred in 95.8%; the most common were headache (58.3%; all grade 1–2) and fatigue (54.2%; all grade 1–2), followed by IRRs (41.7%; grade ≥3, 8.3%), neutropenia (37.5%; grade ≥3, 20.8%), and nausea (33.3%; grade ≥3, 8.3%) (**Table 2**). Clinical symptoms for IRRs included flushing (33.3%), nausea (25.0%), and chills (25.0%). Drug-related TEAEs were reported in 95.8% of patients and included headache and fatigue in 41.7% of patients each, neutropenia in 37.5%, and thrombocytopenia in 29.2%. Grade ≥3 TEAEs occurred in 66.7% of patients; grade ≥3 TEAEs that occurred in more than one patient were neutropenia (20.8%), pain (12.5%), nausea (8.3%), and IRRs (8.3%). SAEs were reported in 33.3% of patients, with the most common being IRRs in 12.5%. One patient discontinued treatment with modakafusp alfa and pembrolizumab due to encephalitis in phase II.

Overall, there were five deaths during the study: three in phase Ib (all disease-related) and two in phase II (one due to gastrointestinal hemorrhage and one for unknown reasons).

### Efficacy

In phase Ib, among the 15 evaluable patients, four patients had a confirmed best response of stable disease (SD), including one patient with cutaneous melanoma in the 0.75 mg/kg cohort, who had a target lesion reduction from baseline of 23%.

**Figure 1:**
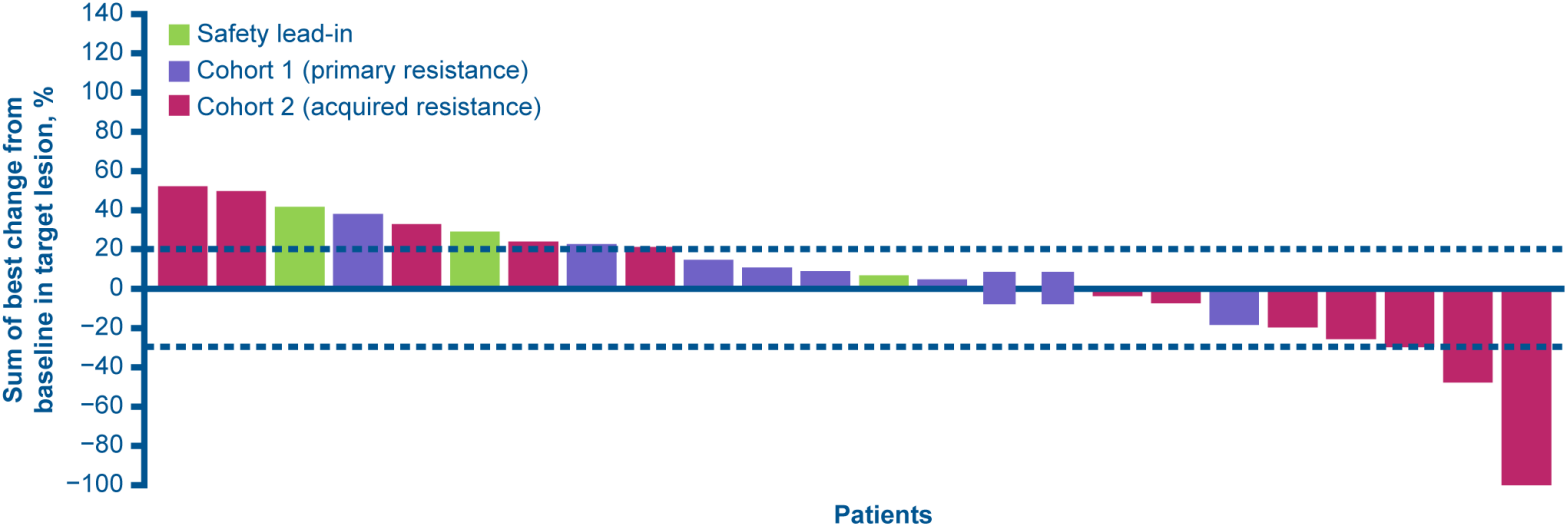
Percentage change from baseline in target lesion reduction in phase II (response-evaluable analysis set). Patients displaying both positive and negative values had a best percent change of 0% in target lesion reduction.

In phase II, among 24 response-evaluable patients, one had a confirmed complete response (CR) and one patient had a confirmed partial response (PR), both in cohort 2, whereas seven had a best response of SD (one in the safety lead-in, and three in each cohort; **Table 3**). Seven patients in cohort 2 had a target lesion reduction (**Figure 1**). The CR and PR lasted for 17 and 12 treatment cycles, respectively (**Supplementary Figure S1**).

**Table 3:** Summary of best overall response (response-evaluable analysis set).

Category, n (%)*	Safety lead-in (n=3)	Cohort I (primary resistance) (n=9)	Cohort II (acquired resistance) (n=12)	Phase II total (N=24)
Best overall response				
Complete response	0	0	1 (8.3)	1 (4.2)
Partial response	0	0	1 (8.3)	1 (4.2)
Stable disease	1 (33.3)	3 (33.3)	3 (25.0)	7 (29.2)
Progressive disease	2 (66.7)	5 (55.6)	6 (50.0)	13 (54.2)
Not evaluable	0	1 (11.1)	1 (8.3)	2 (8.3)
ORR^†^	0	0	2 (16.7)	2 (8.3)
95% CI	0.0–70.8	0.0–33.6	2.1–48.4	1.0−27.0
DCR^‡^	1 (33.3)	3 (33.3)	5 (41.7)	9 (37.5)
95% CI	0.8–90.6	7.5–70.1	15.2–72.3	18.8−59.4

*Unless otherwise stated. ^†^ORR was defined as the proportion of patients who achieved a complete response or partial response.

^‡^DCR was defined as the proportion of patients who achieved a complete response, partial response, or stable disease.

CI, confidence interval; DCR, disease control rate; ORR, overall response rate.

Based on the response rate observed in cohorts 1 and 2 in phase II, the study met its criteria for early termination, and the sponsor decided to stop enrollment.

### PK analyses

**Figure 2:**
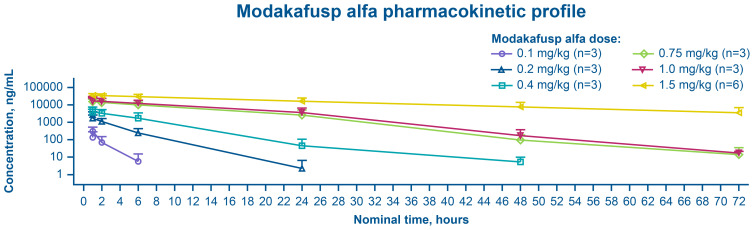
Mean (+standard deviation) serum concentrations of modakafusp alfa versus time following single-dose administration (cycle 1, day 1) in phase Ib (PK analysis set). PK, pharmacokinetics.

The C_max_ of modakafusp alfa was achieved at a median (range) t_max_ of 1 h (1–3), often coinciding with the EOI. After attainment of peak concentrations, serum modakafusp alfa concentrations demonstrated a biphasic decline. The geometric mean apparent t_1/2z_ for cycle 1, day 1 varied, observed at approximately 1.2 h at 0.1 mg/kg (n=3), 2 h at 0.2 mg/kg (n=3), 4 h at 0.4 mg/kg (n=3), 6 h at 0.75 mg/kg (n=3), 7 h at 1.0 mg/kg (n=3), and 16 h at 1.5 mg/kg (n=6) (**Figure 2**). The overall between-patient exposure variability, as determined by percent coefficient of variation values, was generally moderate to high (49% to 145%) for AUC_last_.

Based on the dose-normalized AUC_last_, the exposures of modakafusp alfa increased in a greater than dose-proportional manner in the 0.1- to 1.5-mg/kg dose range, consistent with a saturable target-mediated elimination of modakafusp alfa.

Pembrolizumab PK was comparable with that reported in the literature.

### Immunogenicity

**Figure 3:**
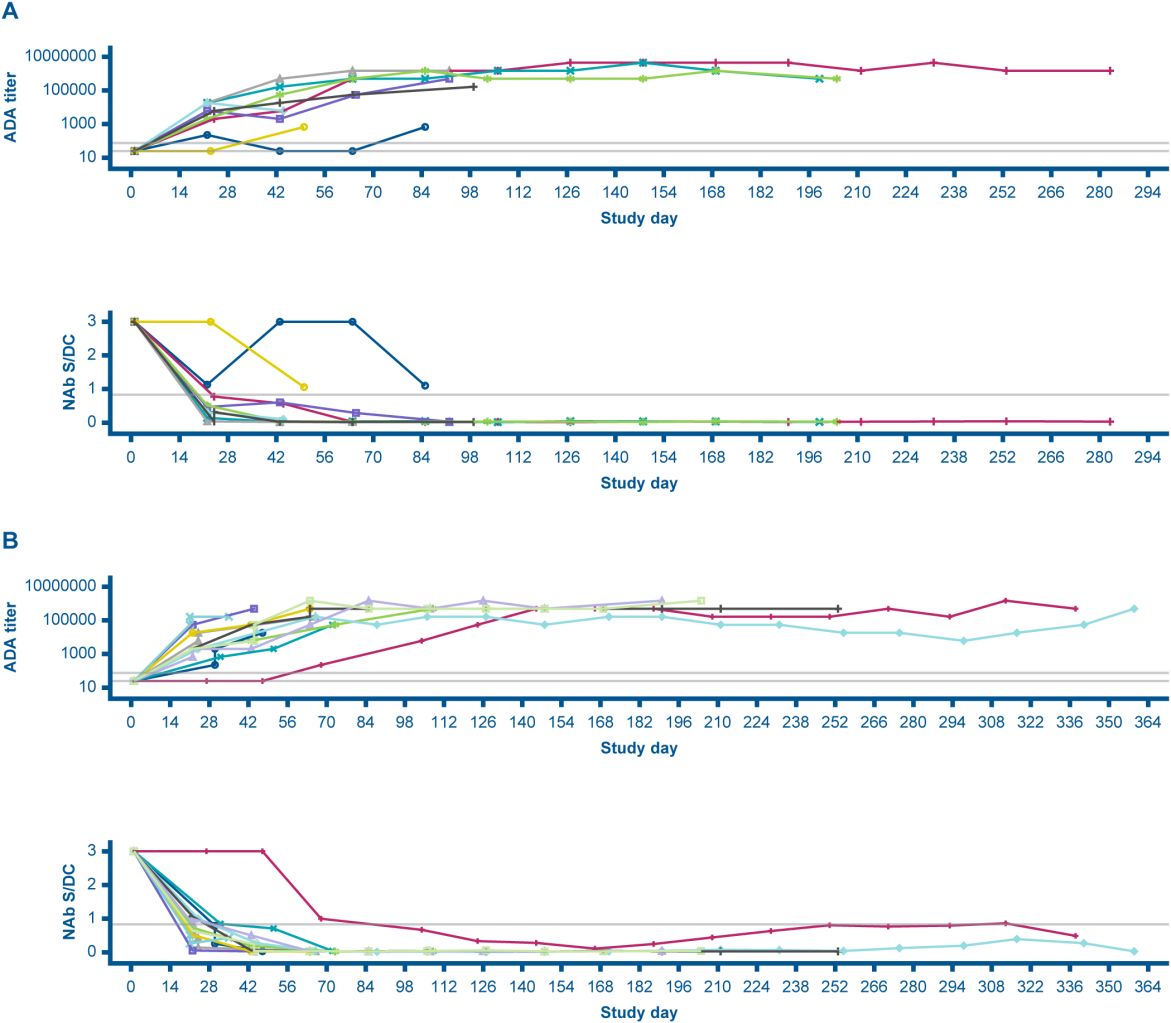
Individual overlay ADA titer and NAb S/DC ratio over time in cohort I **(A)** and cohort II **(B)**. Each line represents the data for an individual patient. NAb S/DC for ADA-negative samples was set to 3 in this plot. ADA, antidrug antibody; NAb, neutralizing antidrug antibody; S/DC, signal-to-drug concentration ratio.

**Figure 4:**
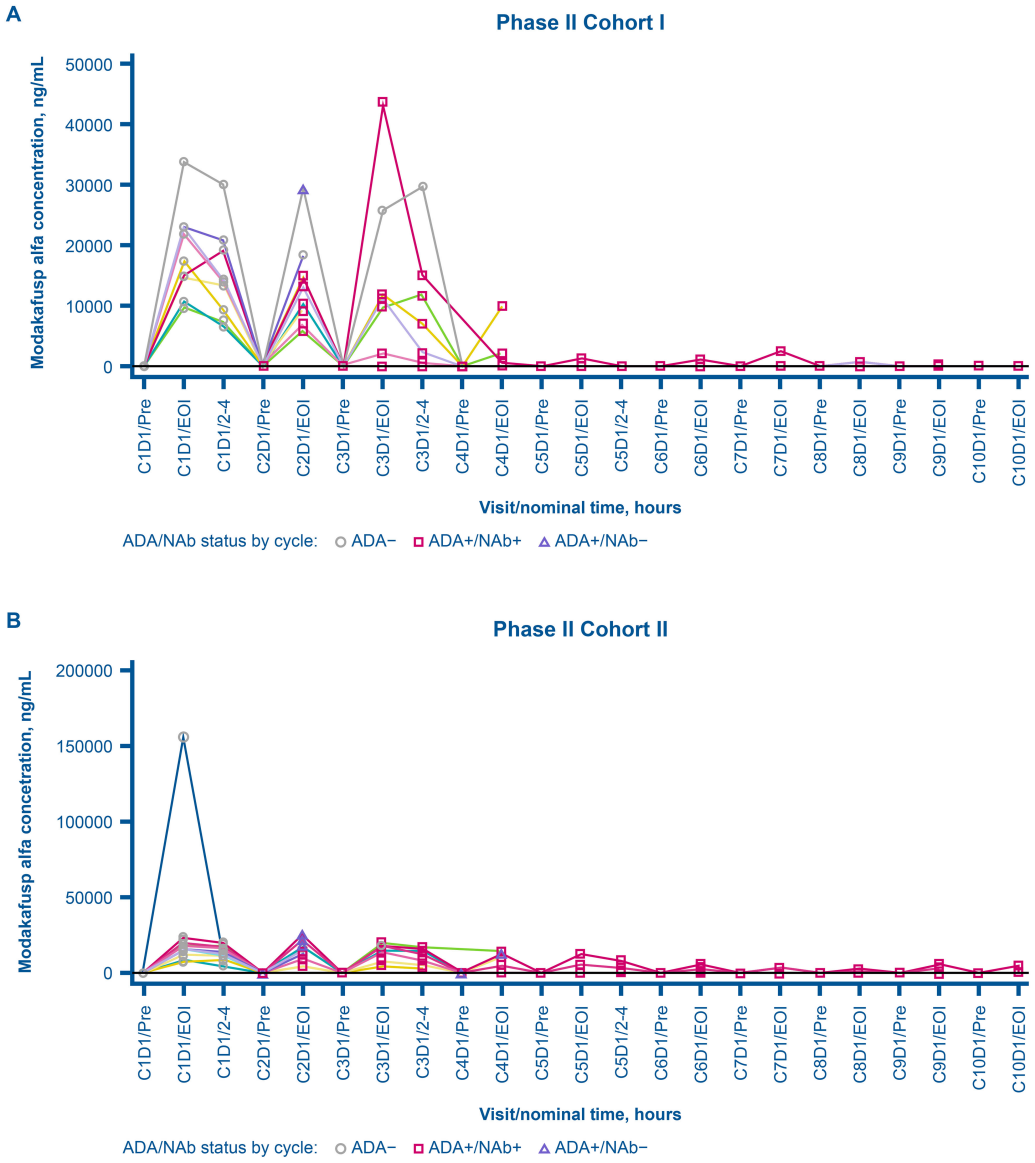
Individual overlay serum concentration of modakafusp alfa versus time by ADA and NAb status for cohort I **(A)** and cohort II **(B)** in phase II dose expansion. Each line represents the data for an individual patient. ADA, antidrug antibody; C, cycle; D, day; EOI, end of infusion, NAb, neutralizing antidrug antibody; Pre, pre-dose.

In phase Ib, among the 17 patients in the immunogenicity-evaluable population, one (5.9%) was ADA-positive at baseline, whereas all were ADA-positive posttreatment. In phase II, of the 22 patients in the immunogenicity-evaluable population, none were ADA-positive at baseline, whereas all 22 patients were ADA-positive following treatment (**Table 4**). No dose response on the ADA incidence and titer was observed. **Figure 3** depicts the dynamics of ADA titers and NAb S/DC ratio over time during the expansion phase. The first incidence of posttreatment ADA was reported as early as cycle 2, day 1 (earliest posttreatment sampling) in both phases Ib and II and the ADA titers tended to increase with duration of treatment and peaked around cycles 4 to 7 in individual patients (**Figures 3A, B**). **Figure 4** presents the overlay of PK concentration over time, stratified by ADA status for cohort I and cohort II. As observed, substantial reductions in the EOI serum concentration of modakafusp alfa, representing the peak PK concentration following each intravenous administration, were noted at later cycles when ADA titers peaked, typically at or after cycle 4 in individual patients (**Figures 4A, B**).

**Table 4:** Summary of patients with ADAs and NAbs at baseline and following treatment with modakafusp alfa in phase Ib and phase II (immunogenicity-evaluable set).

N (%)	Phase Ib dose escalation (N = 17)	Phase II dose expansion (N = 22)
ADA positive at baseline	1 (5.9)	0
NAb positive at baseline	0	0
ADA positive at any posttreatment visit	17 (100)	22 (100)
NAb positive at any posttreatment visit	14 (82.4)	20 (90.9)
Posttreatment ADA positivity (domain specificity)		
Anti-CD38 and IgG4-IFNα2b positive	10 (58.8)	16 (72.7)
Anti-CD38 and IgG4-IFNα2b negative	1 (5.9)	0
Anti-CD38 positive and IgG4-IFNα2b negative	1 (5.9)	2 (9.1)
Anti-CD38 negative and IgG4-IFNα2b positive	5 (29.4)	4 (18.2)

ADA, antidrug antibody; IFNα2b, interferon alpha-2b**;** NAb, neutralizing antibody.

Transient and persistent ADA were reported in phase Ib, but persistent ADA only reported in phase II. The ADA titer ranged from 75 to 1,480,000 in phase Ib, and from 225 to 4,443,000 in phase II. In terms of domain specificity, 58.8% and 72.7% of the immunogenicity-evaluable patients in phases Ib and II, respectively, were reported ADA-positive against both the IgG4-IFNα2b and the anti-CD38 (IgG4) domains. In phase Ib, 29.4%, 5.9%, and 5.9% of patients were reported ADA-positive against IgG4-IFNα2b only, anti-CD38 (IgG4) only, and neither domain, respectively. In phase II, 18.2% and 9.1% of patients were ADA-positive against IgG4-IFNα2b only and anti-CD38 (IgG4) only, respectively.

None of the patients in either phase Ib or II had NAbs at baseline, whereas 14 of 17 patients (82.4%) and 20 of 22 (90.9%), respectively, were NAb-positive after treatment. ADA and NAb positivity were associated with a reduced C_max_, and the degree of reduction tended to increase as the study duration and magnitude of immunogenicity increased.

### Pharmacodynamic analyses

**Figure 5:**
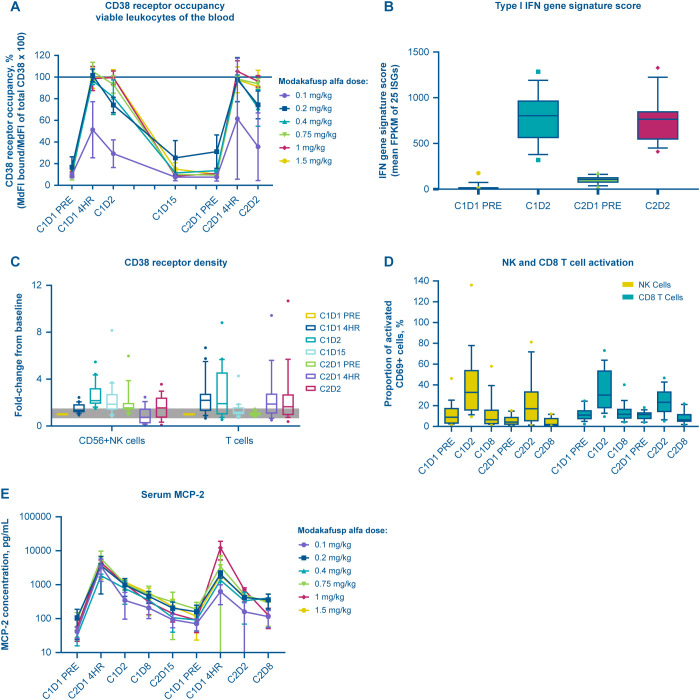
Modakafusp alfa binds to CD38 and induces type I IFN pathway activation in the peripheral blood of solid tumor patients. Line graphs (mean ± standard deviation) and box and whisker plots (whiskers 10–90 percentiles) depict the percent of CD38 receptors on viable leukocytes occupied by modakafusp alfa **(A)**; the fold change in the type I IFN gene signature score, as defined by the average FPKM of 25 ISGs **(B)**; the fold change in CD38 receptor density on NK cells and T cells **(C)**; the percentage of activated NK cells and CD8 T cells, as defined by CD69 expression **(D)**; and serum concentration of MCP-2 **(E)** in peripheral blood samples collected during cycle 1 and cycle 2 of dose escalation. Baseline for all datasets is defined as cycle 1, day 1 pre-dose. C, cycle; D, day; FPKM, fragments per kilobase of transcript per million mapped reads; IFN, interferon; ISG, IFN-stimulated gene; MCP-2, monocyte chemotactic protein-2; mdFL, MyoD family inhibitor; NK, natural killer.

The pharmacodynamic analyses were carried out in samples from all 21 patients treated with modakafusp alfa in phase Ib. Modakafusp alfa was pharmacologically active across all doses analyzed. Modakafusp alfa bound to its target reaching ~100% CD38 receptor occupancy on viable leukocytes at 4 h following administration at ≥0.2 mg/kg (**Figure 5A**). Within 24 h of treatment, peripheral blood analysis showed activation of the IFN pathway, as shown by an elevated type I IFN gene signature score (expressed as average fragments per kilobase per million for the 25-gene IFN-stimulated gene score (21)) (**Figure 5B**). An increase in CD38 receptor density on NK and T lymphocytes and in activation of NK and T cells, as measured by percentage of CD69+ cells, was also seen (**Figures 5C, D**). Elevated serum levels of MCP-2 were observed at 4 h posttreatment (**Figure 5E**).

**Figure 6:**
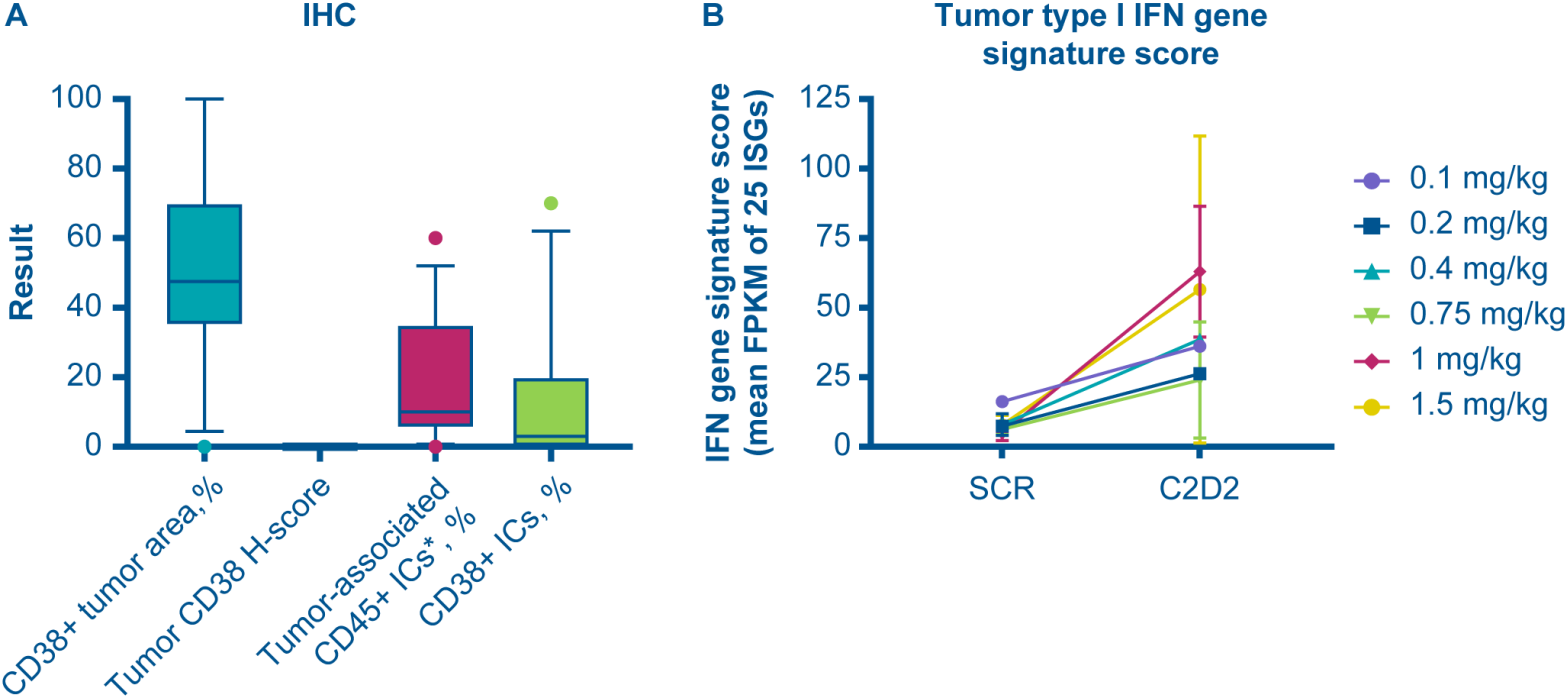
Modakafusp alfa induces type I IFN pathway activation in tumor biopsies of solid tumor patients. Box and whisker plots (10–90 percentile) depict immunohistochemistry results in tumor biopsy sites **(A)**; type I IFN gene signature score, as defined by the average FPKM of 25 ISGs, at screening and C2D2 **(B)** from biopsies collected during dose escalation. CD38+ tumor area % represents percentage of area positive for CD38 staining. Tumor CD38H-score represents intensity of staining in percentage of cells positive (with a low H-score as shown here depicting a very low intensity of CD38 detection in tumor cells). *Tumor-associated CD45+ IC % represents percentage of tumor-associated immune cells detected in the IHC analysis (by CD45 staining). CD38+ IC % represents the percentage of immune cells positive for CD38 staining by IHC. C, cycle; D, day; FPKM, fragments per kilobase of transcript per million mapped reads; IC, immune cells; IFN, interferon; IHC, immunohistochemistry; ISG, IFN-stimulated gene; SCR, screening.

**Figure 7:**
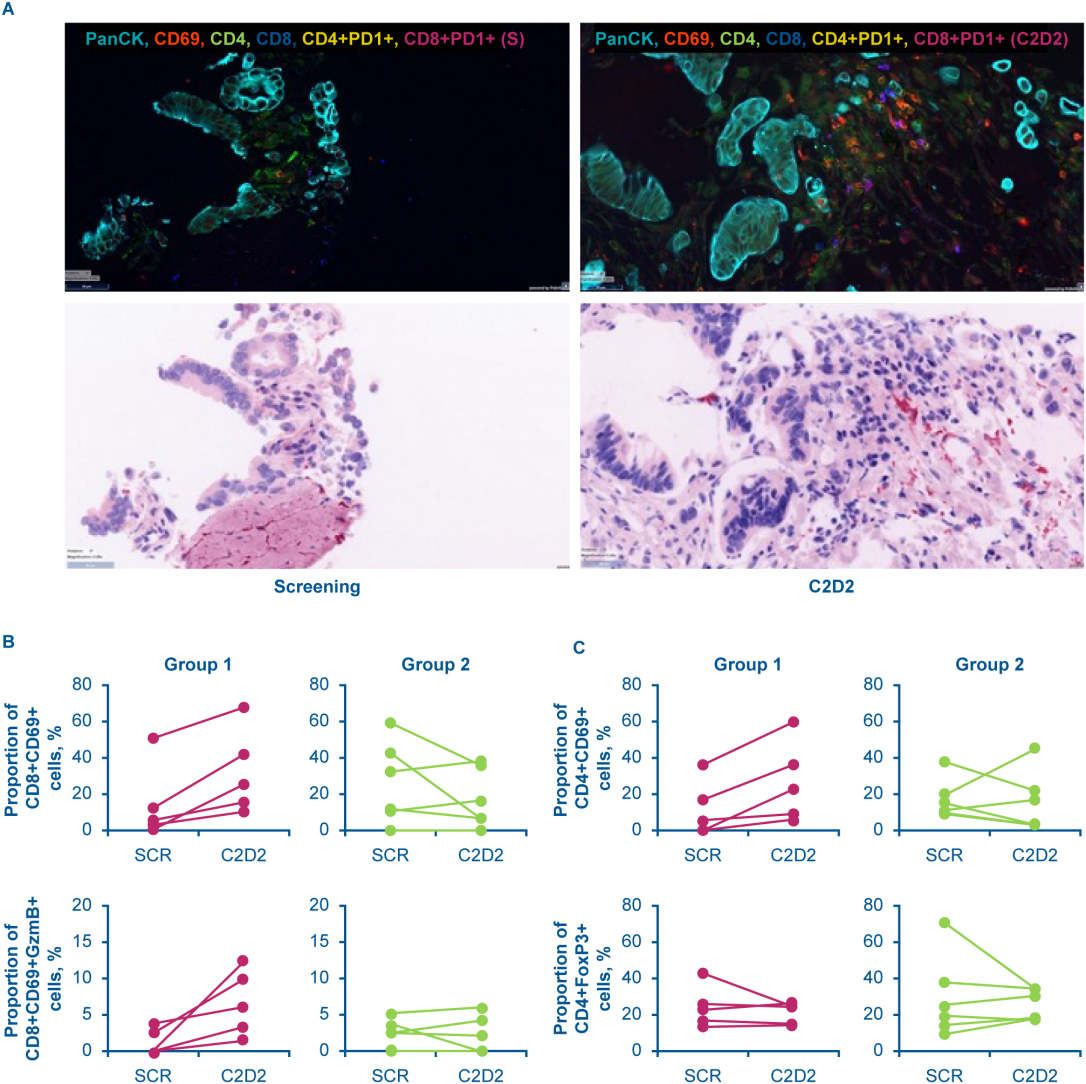
Differential immune cell activation in two subsets of patients with solid tumors with (group 1) and without (group 2) an increase in the percentage of CD38+ lymphocytes in tumor biopsies upon treatment with modakafusp alfa. Representative image from one patient showing IHC and H&E staining **(A)**; line graphs represent change in the percentage of CD69+ CD8 (top panels) and CD4 (bottom panels) lymphocytes **(B)**; line graphs represent change in the percentage of FoxP3+ Tregs (top panels) and CD14+CD16+ myeloid cells (bottom panels) **(C)**. Magenta lines indicate patients that showed an increase in CD38 posttreatment; green lines indicate patients that showed no increase in CD38 posttreatment. Dataset generated from biopsies collected during dose escalation. C, cycle; D, day; FoxP3, forkhead box protein P3; GzmB, granzyme B; H&E, hematoxylin and eosin; IHC, immunohistochemistry; panCK, pan-cytokeratin; PD1, programmed cell death protein 1; SCR, screening.

Levels of CD38 were also analyzed in tumor biopsies, via immunohistochemistry, and showed an average of ~50% positivity for CD38 staining (**Figure 6A**). Similar to the results in peripheral blood, modakafusp alfa led to an elevated type I IFN gene signature score within the tumor biopsies (**Figure 6B**). A detailed analysis of paired tumor biopsies from 11 patients revealed two subgroups defined by differences in CD38 upregulation following modakafusp alfa treatment, which was accompanied by differential intratumoral pharmacodynamic changes (**Table 5**, **Figure 7**). In the subset of patients with an increase in the percentage of CD38+ lymphocytes in response to modakafusp alfa, there were also increases in CD8+ and CD4+ T-cell activation (%CD69) as well as CD8+ T-cell cytotoxic function (%GzB) in the tumor biopsies (**Table 5**, **Figures 7A, B**). Minimal changes in percentages of FoxP3+ Tregs were observed (**Figure 7C**), indicating that modakafusp alfa does not enhance a Treg-mediated suppressive tumor microenvironment. Although differential intratumoral pharmacodynamic responses were observed in a subset of patients, the assessment of correlation with clinical response was not conclusive due to the limited number of paired biopsy samples available for analysis. In addition, there were differences in the sites of biopsy collection pre-dose versus post-dose, which further complicated the analysis.

**Table 5:** Differential immune cell activation in two subsets of patients with solid tumors with (group 1) and without (group 2) an increase in the percentage of CD38+ lymphocytes in tumor biopsies upon treatment with modakafusp alfa.

Group	Patient	Indication	BOR	CD8+			CD4+	
				CD38	CD69	GzmB+	CD38	CD69
Group 1: increase in CD38+ cells	1	Melanoma	SD	+++	+	+++	+++	+
	2	Renal carcinoma	PD	+++	+	++	++	+
	3	Kidney cancer	PD	++	+++	++	No change	++
	4	Other	SD	++	++	++	++	++
	5	Colon cancer	NE	++	+++	+++	No change	++
Group 2: no major change in CD38+ cells	6	Colon adenocarcinoma	SD	+	0	Decrease	+	Decrease
	7	Melanoma	PD	No change	+	+	No change	+
	8	Anal sq. cell carcinoma	SD	No change	0	No change	No change	Decrease
	9	Colon cancer	SD	No change	Decrease	+	No change	Decrease
	10	Colorectal cancer	PD	No change	+	Decrease	No change	++
	11	Rectal cancer	NE	Decrease	Decrease	No change	No change	Decrease

BOR, best overall response; GzmB+, granzyme B positive; NE, not evaluable; PD, progressive disease; SD; stable disease, sq., squamous.

**Figure 8:**
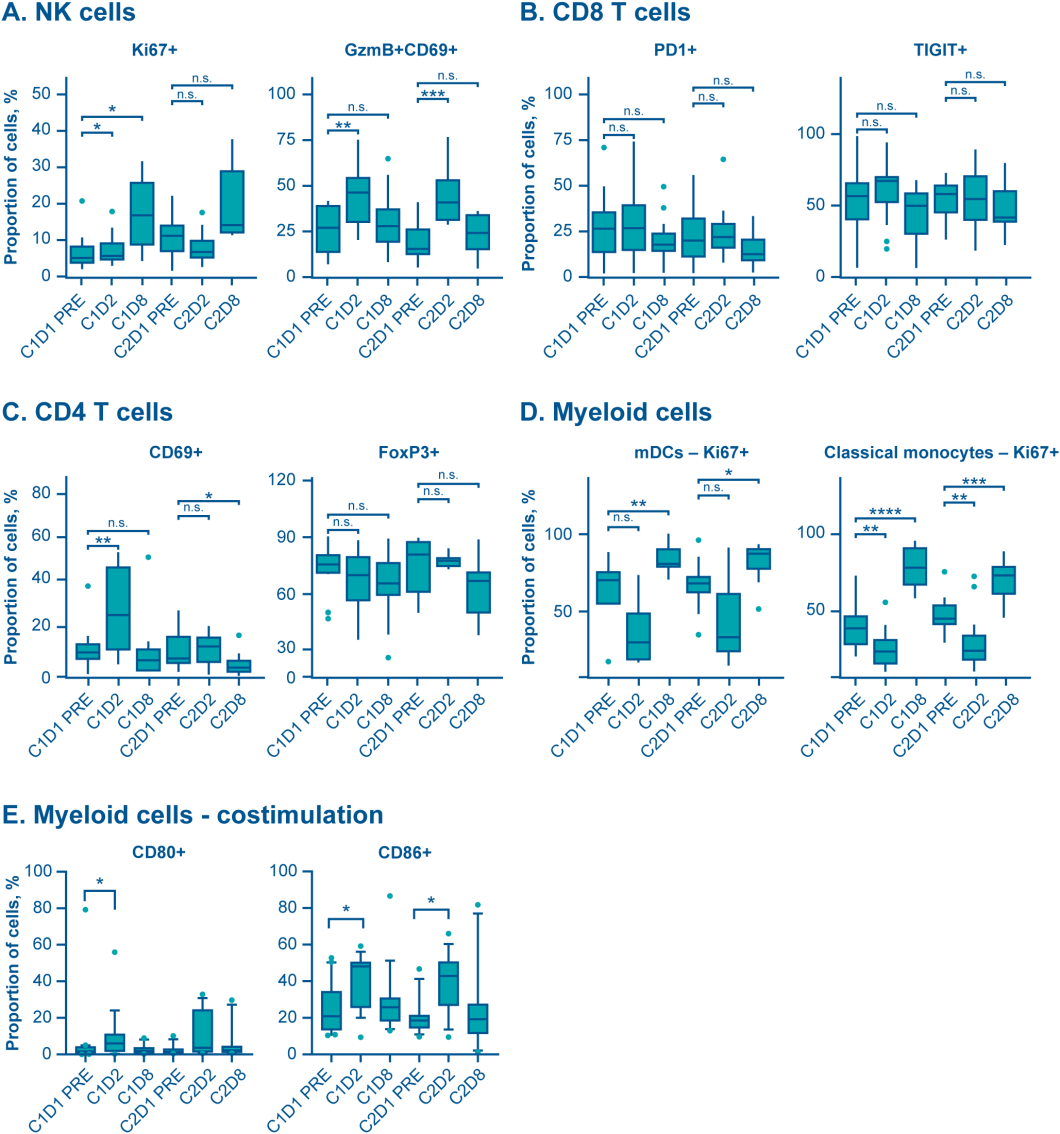
Treatment with modakafusp alfa led to transient enhanced NK cell activation and enhanced myeloid cell response but did not have an effect on T-cell exhaustion or regulatory T-cell levels in peripheral blood. Box and whisker plots depicting pharmacodynamic changes of NK cells **(A)**; CD8 T cells **(B)**; CD4 T cells **(C)**; myeloid cells **(D)**; and costimulatory markers on myeloid cells **(E)**, in samples collected from patients during dose escalation. *p<0.05, **p<0.01, ***p<0.001, ****p<0.0001. Statistical significance was assessed by a two-way ANOVA. C, cycle; D, day; FoxP3, forkhead box protein P3; GzmB, granzyme B; mDC, myeloid dendritic cells; NK, natural killer; n.s., non-significant; PD-1, programmed cell death protein 1; TIGIT, T-cell immunoreceptor with immunoglobulin and ITIM domain.

Modakafusp alfa-mediated immune pharmacodynamic changes in the peripheral blood were seen in most patients regardless of increase in percentage of CD38+ lymphocytes. **Figures 8A, B** shows the effect of modakafusp alfa on NK and CD8+ T cells in peripheral blood of all patients. This was accompanied by increased NK cell proliferation (%Ki67+) and cytolytic activity (%GzmB+%CD69+) (**Figure 8A**) without sustained increase in CD8+ T cell exhaustion markers PD-1 and TIGIT (**Figure 8B**). CD4+ T cells were also activated in response to modakafusp alfa, and there was no increase in circulating FoxP3+ Tregs (**Figure 8C**). Enhanced myeloid cell response, measured by increased proliferation of monocytes and dendritic cells, as well as increased costimulatory markers on monocytes was also observed in the peripheral blood (**Figures 8D, E**).

## Discussion

In this phase Ib/II study, we investigated modakafusp alfa as a single agent in patients with advanced/metastatic solid tumors in phase Ib, and in combination with pembrolizumab in patients with unresectable/metastatic cutaneous melanoma in phase II.

The starting dose and schedule of modakafusp alfa for phase Ib were chosen based on preliminary safety, PK, and pharmacodynamic data from the first-in-human (FIH) study of modakafusp alfa in patients with multiple myeloma (NCT03215030) (22, 11). This suggested that 0.1 mg/kg Q3W was safe, was biologically active, and had the potential to provide clinical benefit. Following the occurrence of DLTs in the 1.5-mg/kg cohort in the present study, an intermediate cohort was started at 1.0 mg/kg. No DLTs were reported in any of the three patients and so enrollment started into the phase II dose expansion safety lead-in cohort to evaluate DLTs with modakafusp alfa at the RP2D in combination with pembrolizumab 400 mg Q6W. No DLTs occurred; therefore, enrollment proceeded for patients with unresectable/metastatic melanoma and primary resistance (cohort 1) or acquired resistance (cohort 2) to one to two prior lines of anti-PD-1 therapy. We were not able to enroll any patients into the anti-PD-1-naïve cohort 3.

In phase II, flu-like symptoms such as headache, fatigue, IRRs, and nausea were among the most common TEAEs reported, most of which were grades 1–2, consistent with findings from the phase Ib KEYNOTE-029 study of pembrolizumab in combination with pegylated IFNα-2b in patients with advanced melanoma or renal cell carcinoma (23). The toxicity profile observed here was generally better than that of traditional adjuvant IFN-α-2b, with which grade ≥3 constitutional and neurological TEAEs are commonly reported (24, 25).

The IRRs reported here may be classified as flu-like side effects typical of IFNα therapy (9); however, most were mild in nature (i.e., included chills and nausea) and did not require hospitalization. Importantly, the combination of modakafusp alfa with pembrolizumab did not result in a higher rate of IRRs in phase II. The reported rates of IRRs in phase Ib (52.4%) were higher than those observed in the FIH study among the 30 patients with multiple myeloma who received modakafusp alfa 1.5 mg/kg Q4W (36.7%) (11). This could be due to the immunosuppressive nature of multiple myeloma or the chronic usage of corticosteroids for multiple myeloma treatment. However, the number of patients in the present study is very low for any comparisons or conclusions to be drawn.

In terms of efficacy, the only two objective responses were observed in cohort 2 (acquired resistance), whereas there was no response in cohort 1 (primary resistance). As CD38 upregulation has been considered a possible mechanism of acquired resistance to CPI therapy in patients with melanoma (8), it is possible that greater levels of CD38 expression are a prerequisite for modakafusp alfa activity in this setting. However, in the multiple myeloma study, no relationship was found between the level of CD38 expression and ORR (11). Nevertheless, due to the low ORR rates observed in phase 2 in the present study, the criteria for early termination were met and the sponsor closed the study and discontinued modakafusp alfa development in the melanoma setting.

Immunogenicity analyses revealed that all immunogenicity-evaluable patients in phases Ib and II were positive for ADA following treatment with modakafusp alfa. This was associated with reduced peak PK concentration at later cycles when ADA titers tended to plateau. However, a limitation of the current PK assay is that high-titer ADA can interfere with PK measurements. In addition, NAbs were reported in 82.4% and 90.9% of patients in phases Ib and II, respectively, which was associated with a reduction in drug pharmacological effect. These rates were much higher than those reported in the phase 1/2 study in patients with relapsed or refractory multiple myeloma (60% posttreatment among patients treated with Q3W and Q4W dosing schedules) (22), which is expected given the immunosuppressive nature of multiple myeloma (26). The high rate of ADA and NAbs observed in the present study, and associated lower drug exposure, could have in part contributed to the low response rates seen particularly in patients who developed high-titer ADA and high NAb activity. Indeed, exposure–response analyses of modakafusp as a single agent in patients with multiple myeloma revealed a positive correlation between exposure and response to treatment (27). Due to the limited number of responders in the present study, exposure–response studies could not be conducted. Nevertheless, while substantial reductions in C_max_ were observed at later cycles in ADA-positive patients, particularly for patients who developed higher ADA titers, the observed C_max_ reduction may be confounded by interference of high-titer ADA on the modakafusp alfa unbound PK assay. A more ADA-tolerant PK assay may be developed in the future to overcome these challenges. Furthermore, although ADA may have contributed to reduced efficacy following decreased exposure in individual patients, the lack of efficacy might also be attributed to the mechanism of action of modakafusp alfa itself. Solid tumors typically exhibit lower or heterogeneous CD38 expression compared with multiple myeloma, potentially limiting the efficacy of this targeted-based mechanism of action over non-targeted IFN.

Pharmacodynamic biomarker data from this phase Ib/II trial in patients with advanced solid tumors demonstrated that modakafusp alfa enhances innate and adaptive immune activation in peripheral blood and within tumors. Peripheral blood changes included activation of the IFN pathway, as shown by an elevated type I IFN gene signature score, with changes observed in genes reported to be related to both acute and chronic IFN signaling activity in solid tumors (21, 28–30). Although chronic type I IFN signaling has been associated with T-cell exhaustion, reduced CD8^+^ T-cell function, and resistance to checkpoint inhibitors (31), the Q3W dosing schedule combined with median (range) t_max_ of 1 h (1–3) of modakafusp alfa, potentially avoids chronic exposure. This is further reflected in the elevated IFN gene signature, which returned to baseline before the start of cycle 2. Nevertheless, we have not assessed whether chronic IFN signaling had an impact on the outcomes observed in this study.

Our pharmacodynamic results are consistent with the expected mechanism of action of modakafusp alfa and with data from the phase I/II and phase II trials in patients with multiple myeloma (11, 32). The presence of a differential intratumoral pharmacodynamic response in a subset of patients with elevated CD38+ cells highlights that tumor-intrinsic factors may play a role in the activity of modakafusp alfa in solid tumors. Nevertheless, the correlative analysis was inconclusive due to the small number of paired biopsy samples and the added implication of different collection sites for pre- and posttreatment biopsies. Importantly, immune pharmacodynamic changes in the peripheral blood were seen in most patients with some differences identified between the two patient subsets defined in the biopsy analysis. The subset of patients with enhanced immune pharmacodynamics in response to modakafusp alfa may have had a greater baseline propensity for immune cell activation (i.e., lower numbers/level of exhausted cells) or higher baseline levels of intratumoral CD38 expression. However, neither of these hypotheses have been tested/analyzed due to limitations of the assay as well as availability of samples.

Taken together, these data show that modakafusp alfa induces innate and adaptive immune responses, supporting its hypothesized mechanism of action in patients with advanced solid tumors. High immunogenicity and/or the potentially limited effect of the IFN mechanism of action for the treatment of solid tumors may have contributed to the low efficacy in these patients.

## Data Availability

The datasets, including the redacted study protocols, redacted statistical analysis plans, and individual participant data supporting the results of the completed study will be made available after the publication of the final study results within 3 months from initial request to researchers who provide a methodologically sound proposal. The data will be provided after its de-identification, in compliance with applicable privacy laws, data protection, and requirements for consent and anonymization.
