# Supplementary material for: A phase Ib/II study of modakafusp alfa alone and in combination with pembrolizumab in patients with advanced or metastatic solid tumors
Source: Front Oncol. 2025 Dec 8;15:1620987. doi: 10.3389/fonc.2025.1620987 (PMC12722991; doi:10.3389/fonc.2025.1620987)
Supplement: Supplementary file 1 [file DataSheet1.docx]

# SUPPLEMENT A phase Ib/II study of modakafusp alfa alone and in combination with pembrolizumab in patients with advanced or metastatic solid tumors

David Gill, Charles L Cowey, Gregory A Daniels, David Sommerhalder, Raghad Abdul-Karim, John M. Kirkwood, Joanna Kolodney, Inderjit Mehmi, Rachel Roberts-Thomson, James Strauss, Sajeve Thomas, Eric Whitman, Yan Xing, Meredith McKean, Sabrina Collins, Cheryl Li, Gurpanna Saggu, Tian Chen, Shining Wang, Marina Lewis, Xavier Parot, and Melissa Johnson

## SUPPLEMENTARY METHODS

**Full inclusion and exclusion criteria**

*Inclusion criteria*

Each patient must have met all the following inclusion criteria to be enrolled in the study:

1. Adult patients aged ≥18 years.

2. Eastern Cooperative Oncology Group performance status of 0–1.

3. Life expectancy >12 weeks according to investigator’s judgment.

4. Phase Ib dose escalation: Eligible patients must have histologically confirmed advanced (locoregionally recurrent, not amenable to curative therapy) or metastatic solid tumors.

5. Measurable disease per Response Evaluation Criteria in Solid Tumors version 1.1. At least one target lesion amenable for biopsy was required for enrollment in phase Ib. A minimum of one target lesion for response assessment was required for enrollment in phase II. A separate lesion amenable for biopsy was required for enrollment in phase II for cohorts 1 and 2 post-futility analysis and for all patients (safety lead-in and expansion) with subgroup III melanoma.

6. Phase Ib dose escalation: Patients with histologically confirmed advanced locally (locoregionally recurrent, not amenable to curative therapy) or metastatic solid tumors.

Phase II dose expansion: The combination cohorts, including patients in the safety lead-in phase, will enroll patients with unresectable/metastatic melanoma in the following subgroups:

1. Unresectable/metastatic histologically confirmed cutaneous melanoma with primary resistance to ≥2 prior lines of anti-programmed cell death (PD-1)-containing treatments in the metastatic setting.

2. Unresectable/metastatic histologically confirmed cutaneous melanoma with acquired resistance to ≥2 prior lines of anti-PD-1-containing treatments in the metastatic setting.

3. Unresectable/metastatic histologically confirmed cutaneous melanoma naïve to prior anti-PD-1-containing treatments in the metastatic setting.

1. Patients with BRAF V600E mutant melanoma may have received prior BRAF inhibitor therapy.
2. For cohorts 1 and 2, there is no limitation of total number of prior line(s) of therapy, but the number of prior line(s) containing anti-PD-1 must be ≤2 in the metastatic setting.
3. For the expansion cohort 3, patients who received an anti-PD-1 treatment in the adjuvant setting must have completed that treatment at least 6 months prior to enrollment and must not have progressed on the anti-PD-1-adjuvant treatment.
4. Primary resistance is defined as a best response of progressive disease or stable disease <6 months to an anti-PD-1 alone or in combination with other agents (i.e., CTLA4) in the initial anti-PD-1-containing treatment.
5. Acquired resistance defined as a progression following a best response of complete response, partial response, or stable disease >6 months prior to an anti-PD-1 alone or in combination with other agents (i.e., CTLA4).

7. Reproductively female patients who:

1. Were postmenopausal for ≥2 years before the screening visit, OR
2. Were surgically sterile, OR
3. If they were of childbearing potential, due to unknown risks and potential harm to an unborn child/infant, must agree to the following:

- Practiced one highly effective method of contraception and one additional effective (barrier) method at the same time, from the time of signing the informed consent through 7 days after the last dose of modakafusp alfa or 4 months after the last dose of pembrolizumab, whichever is longer, OR
- Agreed to practice true abstinence, when this is in line with the preferred and usual lifestyle of the patient. (Periodic abstinence [e.g., calendar, ovulation, symptothermal, post-ovulation methods], withdrawal, spermicides only, and lactational amenorrhea are not acceptable methods of contraception. Female and male condoms should not be used together).
- Agreed not to donate an egg or eggs (ova) or breastfeed a baby during the study and through 7 days after the last dose of modakafusp alfa or 4 months after the last dose of pembrolizumab, whichever is longer.

8. Reproductively male patients, even if surgically sterilized (i.e., status post-vasectomy), who:

1. Agreed to practice effective barrier contraception during the entire study treatment period and through 7 days after the last dose of modakafusp alfa (no restriction for pembrolizumab), OR
2. Agreed to practice true abstinence, when this is in line with the preferred and usual lifestyle of the patient. (Periodic abstinence [e.g., calendar, ovulation, symptothermal, post-ovulation methods], withdrawal, spermicides only, and lactational amenorrhea are not acceptable methods of contraception. Female and male condoms should not be used together).
3. Agreed not to donate sperm during the study and through 7 days after the last dose of modakafusp alfa (no restriction for pembrolizumab).

9. Voluntary written consent must be given before performance of any study-related procedure not part of standard medical care, with the understanding that consent may be withdrawn by the patient at any time without prejudice to future medical care.

10. Adequate bone marrow reserve and renal and hepatic function based on the following laboratory parameters:

- 1. Absolute neutrophil count (ANC) ≥1.0 × 10^9^/L, platelet count ≥75.0 × 10^9^/L, and hemoglobin ≥80 g/L without growth factor or transfusion support for ANC and platelets in the preceding 2 weeks.
  2. Total bilirubin ≤1.5 times the upper limit of normal (ULN).
  3. Serum alanine aminotransferase or aspartate aminotransferase ≤3.0 times the ULN (<5 times the ULN if liver enzyme elevations are due to liver metastases).
  4. Creatinine <1.5 times the ULN or estimated glomerular filtration rate ≥30 mL/min/1.73 m^2^ using the Modification of Diet in Renal Disease (Levey et al. 2006) or Chronic Kidney Disease Epidemiology Collaboration (Levey et al. 2009) equations.

11. Patients must agree to the applicable biopsy requirements as detailed in the schedule of events.

*Exclusion Criteria*

Patients meeting any of the following exclusion criteria were not enrolled in the study:

1. Treatment with any standard of care or investigational anticancer drug within 28 days or 5 half-lives before administration of modakafusp alfa, whichever comes first. The washout period is 3 weeks for previous major surgery, 2 weeks for previous radical radiation (including chemoradiation and whole-brain radiation), and 5 days from last dose for focal radiation for symptomatic metastases.

2. Persistent toxicity from previous treatments that had not resolved to National Cancer Institute Common Terminology Criteria for Adverse Events version 5 (NCI CTCAE v.5) grade 1 prior to administration of modakafusp alfa, except for alopecia, grade 2 neuropathy, grade 2 asthenia/fatigue, or autoimmune endocrinopathies with stable replacement therapy.

3. History of any of the following ≤6 months before the first dose of modakafusp alfa: New York Heart Association grade 3 or 4 congestive heart failure, unstable angina, myocardial infarction, unstable symptomatic ischemic heart disease, any ongoing symptomatic cardiac arrhythmias grade >2, pulmonary embolism, symptomatic cerebrovascular events, or any other serious cardiac condition (e.g., symptomatic pericardial effusion or restrictive cardiomyopathy). Chronic, stable atrial fibrillation on stable anticoagulant therapy, including low molecular-weight heparin, is allowed.

4. Baseline QTcF >480 msec (grade ≥2), history of congenital long QT syndrome, or torsades de pointes.

5. History of immune-related adverse events (AEs) related to treatment with prior anti-PD-1/PD-L1 that required treatment discontinuation (phase II).

6. Psychiatric illness/social circumstances that would limit compliance with study requirements and substantially increase the risk of AEs or compromised ability to provide written informed consent.

7. History of uncontrolled brain metastasis or previously treated metastases receiving corticosteroid dose >20 mg/day of prednisone equivalent at the time of receiving the first dose of modakafusp alfa.

*Note:* Patients with carcinomatosis meningitis or leptomeningeal disease are excluded, regardless of clinical stability.

8. Patients with uveal (ocular) or mucosal melanoma (phase II).

9. Patients with acral lentiginous melanoma were excluded in phase II except for the safety lead-in phase.

10. Ongoing or active infection.

11. Known history of HIV infection or any other relevant congenital or acquired immunodeficiency.

12. Known hepatitis B (HBV) surface antigen seropositive or detectable hepatitis C infection viral load. Note: Patients with a positive HBV core antibody can be enrolled but must have an undetectable HBV viral load.

13. Autoimmune disease requiring systemic immunosuppressive therapy. Patients with immune-mediated endocrine deficiency from previous therapy with stable hormone replacement were exceptions.

14. History of severe allergic or anaphylactic reaction to recombinant proteins or excipients used in modakafusp alfa or pembrolizumab formulation.

# SUPPLEMENTARY FIGURES

## Supplementary Figure S1. Gating strategy of the CD38 RO/RD assay


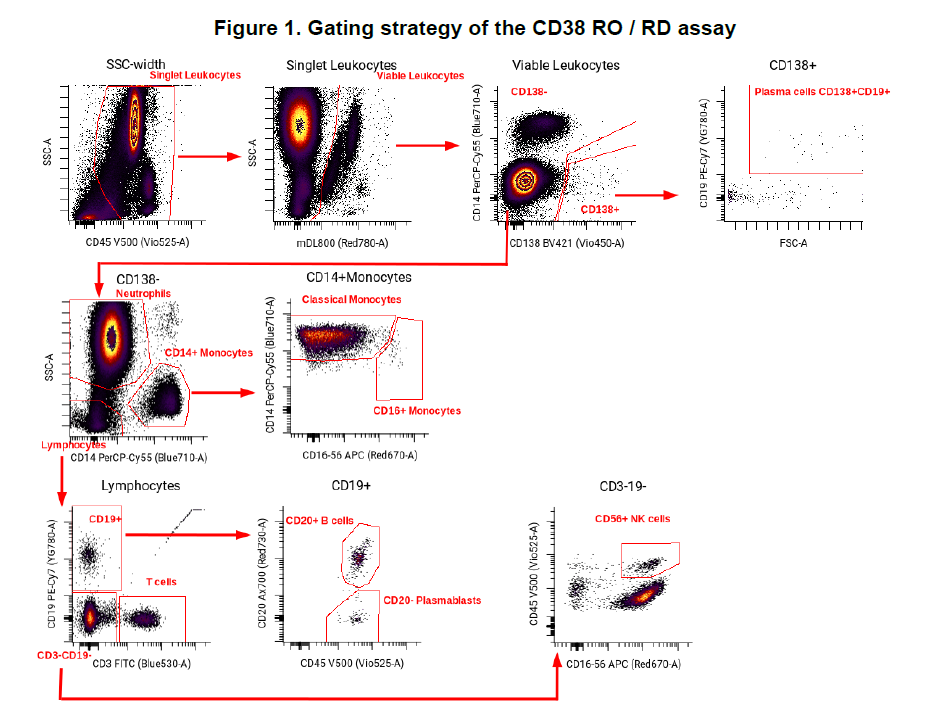


RD, receptor density; RO, receptor occupancy.

## Supplementary Figure S2. Gating strategy of the CyTOF Immunophenotyping Assay

##
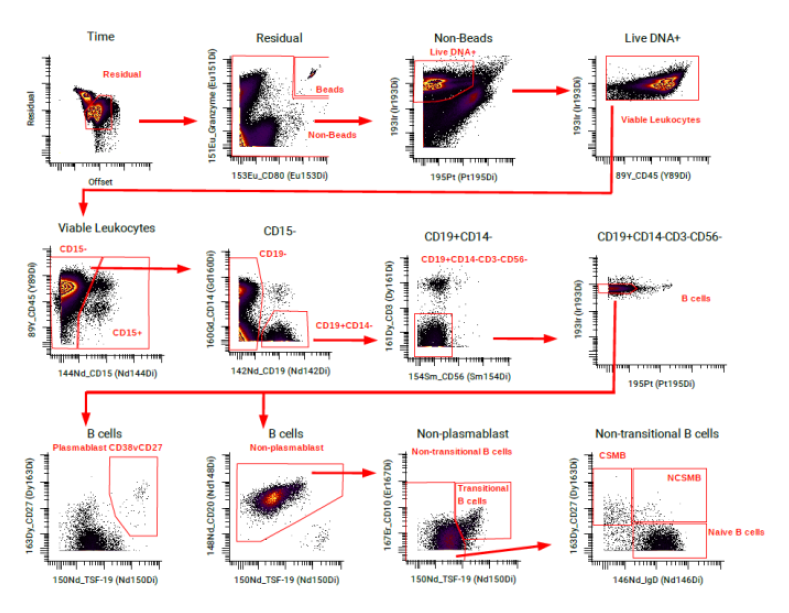


##
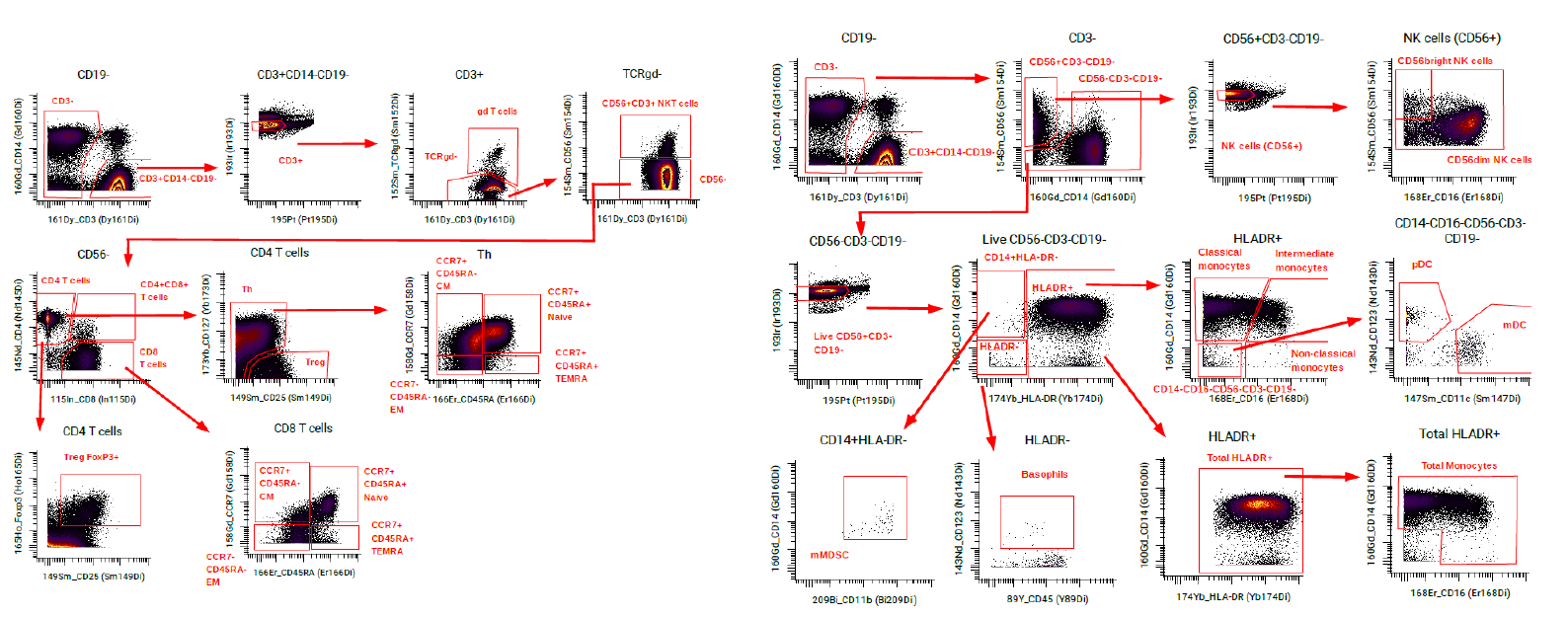


## CyTOF, cytometry by time of flight.

## Supplementary Figure S3. Swimmer plot of confirmed best overall response over time in phase II safety lead-in and dose expansion study (safety analysis set)


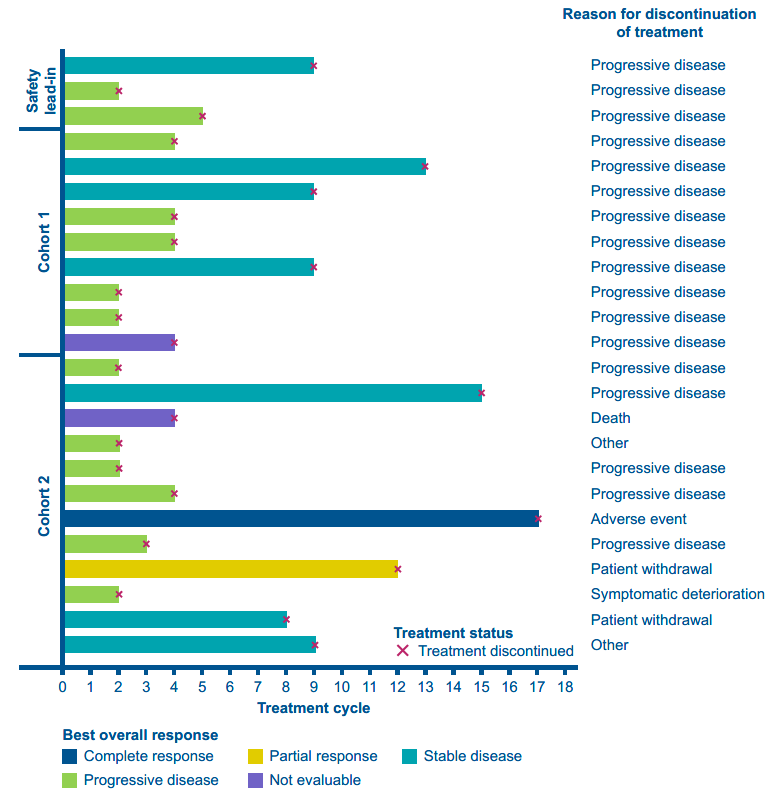


# SUPPLEMENTARY TABLES

## Supplementary Table S1. Surface staining antibodies for the CD38 RO/RD assay

| **Target** | **Fluorochrome** | **Clone** | **Manufacturer or Supplier** | **Catalog Number*** |
| --- | --- | --- | --- | --- |
| CD45 | V500 | HI30 | BD Biosciences | 325622 |
| CD14 | PerCP-Cy5.5 | HCD14 | BioLegend | 300440 |
| CD3 | FITC | UCHT1 | BioLegend | 302012 |
| CD16 | APC | B73.1 | BioLegend | 360706 |
| CD56 | APC | HCD56 | BioLegend | 318310 |
| CD19 | PE-Cy7 | HIB19 | BioLegend | 302216 |
| CD20 | AF700 | 2H7 | BioLegend | 302322 |
| CD138 | BV421 | MI15 | BioLegend | 356516 |
| CD38 | N/Ap | TEV-48573 | Modakafusp alfa provided by Takeda | N/Ap |
| Human IgG4 | PE | HP6025 | Southern Biotech | 9200-09 |

*Catalog number may vary depending on format.

RD, receptor density; RO, receptor occupancy.

**Supplementary Table S2.** Antibodies for the CyTOF Immunophenotyping Assay

| **Marker** | **Metal Isotope** | | **Clone** | **Vendor** | **Catalog  Number*^i^*** |
| --- | --- | --- | --- | --- | --- |
|  | **Name** | **Symbol** |  |  |  |
| CD45 | Yttrium-89 | **89_Y_** | HI100 | Fluidigm | 3089003B |
| Ki-67 | Indium-113 | **^113^In** | B56 | BD | 556003 |
| CD8 | Indium-115 | **^115^In** | RPA-T8 | Biolegend | 301074 |
| CD19 | Neodymium-142 | **^142^Nd** | HIB19 | Fluidigm | 3142001B |
| CD123 (IL-3R) | Neodymium-143 | **^143^Nd** | 6H6 | Fluidigm | 3142001B |
| CD15 | Neodymium-144 | **^144^Nd** | W6D3 | Biolegend | 323002 |
| CD4 | Neodymium-145 | **^145^Nd** | RPA-T4 | Biolegend | 300571 |
| IgD | Neodymium-146 | **^146^Nd** | IA6-2 | Biolegend | 348202 |
| CD11c | Samarium-147 | **^147^Sm** | Bu15 | Biolegend | 337202 |
| CD20 | Neodymium-148 | **^148^Nd** | 2H7 | Biolegend | 302302 |
| CD25 | Samarium-149 | **^149^Sm** | 2A3 | Fluidigm | 3149010B |
| CD38 | Neodymium-150 | **^150^Nd** | TSF-19 | Sponsor provided | N/Ap |
| TCRgd | Samarium-152 | **^152^Sm** | B1 | Biolegend | 331236 |
| CD56 | Samarium-154 | **^154^Sm** | NCAM16.2 | Biolegend | 559043 |
| CCR7 | Gadolinium-158 | **^158^Gd** | G043H7 | Biolegend | 353222 |
| CD14 | Gadolinium-160 | **^160^Gd** | RMO52 | Beckman | IM0643 |
| CD3 | Dysprosium-161 | **^161^Dy** | UCHT1 | Biolegend | 300438 |
| CD27 | Dysprosium-163 | **^163^Dy** | M-T271 | Biolegend | 356401 |
| CD69 | Dysprosium-164 | **^164^Dy** | FN50 | Biolegend | 310902 |
| CD45RA | Erbium-166 | **^166^Er** | HI100 | Fluidigm | 3166031B |
| CD10 | Erbium-167 | **^167^Er** | HI10a | Biolegend | 312202 |
| CD16 | Erbium-168 | **^168^Er** | 3G8 | Biolegend | 302014 |
| CD127 | Ytterbium-173 | **^173^Yb** | A019D5 | Biolegend | 351302 |
| HLADR | Ytterbium-174 | **^174^Yb** | L243 | Biolegend | 307648 |
| CD11b | Bismuth-209 | **^209^Bi** | ICRF44 | Fluidigm | 3209003B |
| HLA-A,B,C | Praseodymium-  141 | **^141^Pr** | W6/32 | Biolegend | 311402 |
| Granzyme B | Europium-151 | **^151^Eu** | QA16A02 | Biolegend | 372202 |
| CD80 | Europium-153 | **^153^Eu** | 2D10 | Biolegend | 305212 |
| CD154  (CD40L) | Gadolinium-155 | **^155^Gd** | 24-31 | Biolegend | 310802 |
| PD-L1 | Gadolinium-156 | **^156^Gd** | 29E.2A3 | Selleck Chem | A2013 |
| CTLA-4 | Terbium-159 | **^159^Tb** | L3D10 | Biolegend | 349902 |
| CD86 | Dysprosium-162 | **^162^Dy** | IT2.2 | Biolegend | 305410 |
| PD-1 | Thulium-169 | **^169^Tm** | EH12.2h7 | Biolegend | 329902 |
| CD138 | Erbium-170 | **^170^Er** | DL-101 | Biolegend | 352302 |
| TIGIT | Ytterbium-171 | **^171^Yb** | A15153G | Biolegend | 372720 |
| CD83 | Ytterbium-172 | **^172^Yb** | HB15e | Biolegend | 305302 |
| Foxp3 | Holmium-165 | **^165^Ho** | 259D | Biolegend | 320202 |
| IFNg | Lutetium-175 | **175 Lu** | B27 | Biolegend | 506502 |
| CD68 | Ytterbium-176 | **176 Yb** | YI/82A | Biolegend | 33380 |

*Catalog number may vary depending on format.

CyTOF, cytometry by time of flight.

## Supplementary Table S3. Associated reagents, probes and antibodies used in MultiOmyx Hyperplexed immunofluorescence assay of tumor biopsy samples

| **Antibody [Clone]** | **Vendor, Catalog#** |
| --- | --- |
| CD3 [F7.2.38] | Dako, Catalog# M7254 |
| CD4 [EPR6855] | Abcam, ab181724 |
| CD8 [C8/144B] | Dako, Catalog# M7103 |
| CD14 [EPR3652] | Abcam, Catalog# ab209971 |
| CD16 [DJ130c] | Thermo, Catalog# MA1-84008S4 |
| CD20 [EP459Y] | Abcam, Catalog# ab166865 |
| CD38 [SPC32] | Leica, Catalog# NCL-CD38-290 |
| CD45 [2B11+PD7/26] | Dako, Catalog# M0701 |
| CD47 [SP279] | Abcam, Catalog# ab236234 |
| CD56 [MRQ-42] | Cell Marque, Catalog# 156R-OEM0714 |
| CD57 [VC1.1] | Sigma, Catalog# C0678 |
| CD68 [KP1] | BioLegend, Catalog# 916104 |
| CD69 [A-5] | Santa Cruz, Catalog# sc-373798 |
| FOXP3 [206D] | BioLegend, Catalog# 320114 |
| Granzyme B [GrB-7] | Dako, Catalog# M7235 |
| HLA-DR [WR18] | Novus, Catalog# NB100-64358 |
| Ki67 [SP6] | Abcam, Catalog# ab16667 |
| PanCK [AE-1/PCK26]* | Abcam/Sigma, (catalog #ab213135/C5992) |
| PD-1 [EPR4877(2)] | Abcam, ab137132 |
| PD-L1 [28-8]^†^ | Abcam, Catalog# ab205921 |
| PD-L1 [22C3] | Dako, Catalog# M3653 |
| SOX-10 [EP268] | Sigma, Catalog# 383R-16 |

*PanCK is a cocktail that includes AE-1 and PCK-26 antibodies to detect low and high molecular weight cytokeratins.

^†^The incorrect antibody clone [28-8] was initially used for PD-L1, and the correct clone 22C3 was added to the panel.

## Supplementary Table S4. Staining sequence used in the 20-marker panel

| **Round of imaging** | **Cy3 Marker** | **Cy5 Marker** |
| --- | --- | --- |
| **1** | PD-L1 [28-8] | CD57 |
| **2** | PanCK or SOX-10 | CD56 |
| **3** | CD3 | CD47 |
| **4** | CD4 | PD-1 |
| **5** | CD8 | FOXP3 |
| **6** | CD14 | CD45 |
| **7** | CD38 | CD16 |
| **8** | HLA-DR | Ki67 |
| **9** | Granzyme B | CD68 |
| **10** | CD20 | CD69 |
| **11** | - | PD-L1 [22C3] |

## Supplementary Table S5. Patient disposition and treatment exposure

| **Patient disposition** | **Phase 1b  (dose escalation) (n=21)** | **Phase II  (dose expansion) (n=24)** |
| --- | --- | --- |
| Ongoing on-study drug, n (%) | 0 | 0 |
| Discontinued study drug, n (%) | 21 (100) | 24 (100) |
| Progressive disease | 13 (61.9) | 17 (70.8) |
| Adverse event | 3 (14.3) | 1 (4.2) |
| Patient withdrawal | 3 (14.3) | 2 (8.3) |
| Symptomatic deterioration | 0 | 1 (4.2) |
| Death | 0 | 1 (4.2) |
| Other | 2 (9.5) | 2 (8.3) |
| Median duration of treatment, weeks (range) | 6.0 (3.0–33.3) | 12.6 (5.9–51.6) |
| Median number of cycles (range) | 2 (1–11) | 4 (2–17) |
